# Association of Serum Magnesium Levels with Frequency of Acute Exacerbations in Chronic Obstructive Pulmonary Disease: A Prospective Study

**DOI:** 10.1155/2014/329476

**Published:** 2014-11-18

**Authors:** Aziz Gumus, Muge Haziroglu, Yilmaz Gunes

**Affiliations:** ^1^Department of Pulmonary Medicine, Recep Tayyip Erdogan University, 53000 Rize, Turkey; ^2^Cardiology Department, Hisar Intercontinental Hospital, 34375 Istanbul, Turkey

## Abstract

*Background*. The course of chronic obstructive pulmonary disease (COPD) is accompanied by acute exacerbations. The purpose of this study is to determine the association of serum magnesium level with acute exacerbations in COPD (COPD-AE). *Materials and Methods*. Eighty-nine patients hospitalized with COPD-AE were included. Hemogram, biochemical tests, and arterial blood gases were analyzed. Pulmonary function tests were performed in the stable period after discharge. Patients were followed up at 3 monthly periods for one year. *Results*. Mean age of the patients was 70.4 ± 7.8 (range 47–90) years. Mean number of COPD-AE during follow-up was 4.0 ± 3.6 (range 0–15). On Spearman correlation analysis there were significant negative correlations between number of COPD-AE and predicted FEV1% (*P* = 0.001), total protein (*P* = 0.024), globulin (*P* = 0.001), creatinine (*P* = 0.001), and uric acid levels (*P* = 0.036). There were also significant positive correlations between number of COPD-AE and serum magnesium level (*P* < 0.001) and platelet count (*P* = 0.043). According to linear regression analysis predicted FEV1% (*P* = 0.011), serum magnesium (*P* < 0.001), and globulin (*P* = 0.006) levels were independent predictors of number of COPD-AE. *Conclusions*. In this small prospective observational study we found that serum magnesium level during exacerbation period was the most significant predictor of frequency of COPD-AE.

## 1. Introduction

Chronic obstructive pulmonary disease (COPD) is a preventable and treatable disease, generally progressive in nature, characterized by chronic inflammatory response of the airways and lungs to harmful gasses and particles, particularly tobacco and biomass fuel smoke (GOLD 2014) [1]. Acute exacerbations that compromise quality of life, accelerate a decline in respiratory functions, and increase economic costs may occur during the course of stable COPD [2, 3]. COPD exacerbation was defined as an acute worsening of respiratory symptoms (increased dyspnea, increased cough or change in amount, and purulence of sputum) that was beyond normal day-to-day variations of symptoms [1]. COPD acute exacerbation (COPD-AE) frequently appears with respiratory tract infections. It is a significant cause of mortality and morbidity [4].

Few studies have investigated the factors leading to exacerbations. Advanced age, low FEV1%, advanced stage of disease, poor performance status, accompanying anxiety and/or depression, poor quality of life, history of frequent exacerbation, hypercapnia, and prolonged duration of disease have all been identified as factors causing frequent exacerbation [5–8].

Magnesium is involved in such important functions as bronchodilation and contraction in respiratory tract smooth muscles, mast cell stabilization, neurohumoral mediator release, and mucociliary clearance [9]. Magnesium is thought to have a protective effect against chronic respiratory tract diseases. It has been suggested that insufficient magnesium intake through diet may lead to development of asthma and COPD [10]. However, insufficient information is available concerning the effect of magnesium on frequency of COPD-AE. The purpose of this study was to determine the factors associated with COPD-AE and to investigate the effect of magnesium levels on incidence of exacerbations.

## 2. Materials and Methods

The study was performed at the Recep Tayyip Erdogan University Faculty of Medicine Chest Diseases Clinic. Local ethical committee approval and written informed consent of patients were obtained. Ninety-four consecutive patients aged 40 or above, hospitalized with diagnosis of COPD-AE meeting the unit admission criteria [11] between May 2012 and March 2013, were included in the study. Five patients died during 1-year follow-up and were excluded from study. Arterial blood gasses, C-reactive protein (C-RP), hemogram, sedimentation rate, and serum electrolyte concentrations including magnesium, creatinine, uric acid, liver function tests, troponin-I, D-dimer, blood glucose, cholesterol, and triglyceride levels were measured within 24 hours of admission. Pulmonary function tests were performed in the stable period after discharge. Patients were followed up at 3 monthly intervals for one year. Patients experiencing acute exacerbations were treated on either an in- or outpatient basis. Patients requiring intensive care due to acute exacerbations, patients with active cancer, cirrhosis, and acute or chronic kidney or heart failure, and who died during monitoring were excluded from the study.

### 2.1. Statistical Analysis

Statistical analyses were performed on IBM-SPSS (SPSS version 21; SPSS Inc., Chicago, IL, USA) software. Constant variables were expressed as mean ± standard deviation and categorical variables as %. The independent samples *t*-test was used for continuous variables. Chi square test was used to compare categorical variables. Correlations between variables were investigated using Pearson correlation analysis for parametric variables and Spearman correlation analysis for nonparametric variables. Linear regression analysis was performed in order to identify independent factors affecting the number of exacerbations in 1 year. ROC curve analysis was performed in order to reveal the effect of serum magnesium level on exacerbation in 1 year. *P* < 0.05 value was considered statistically significant.

## 3. Results

Mean age of patients with COPD-AE was 70.4 ± 7.8 (range 47–90). Almost all were male (88 male, 1 female). Nineteen (21%) patients were current smokers, 69 (78%) were ex-smoker, and 1 (1%) had never smoked. Pulmonary function tests were predicted FVC%: 54 ± 16, predicted FEV1%: 39 ± 14, and predicted FEV1/FVC%: 56 ± 11. COPD staging was performed on the basis of these results. Very severe COPD was determined in 22 (%25) patients (stage 4), severe in 49 (55%) (Stage 3), and moderate COPD in 18 (20%) (Stage 2). None of the patients in the study group had mild COPD. Demographic characteristics of the patients are given in Table 1.

**Figure 1:**
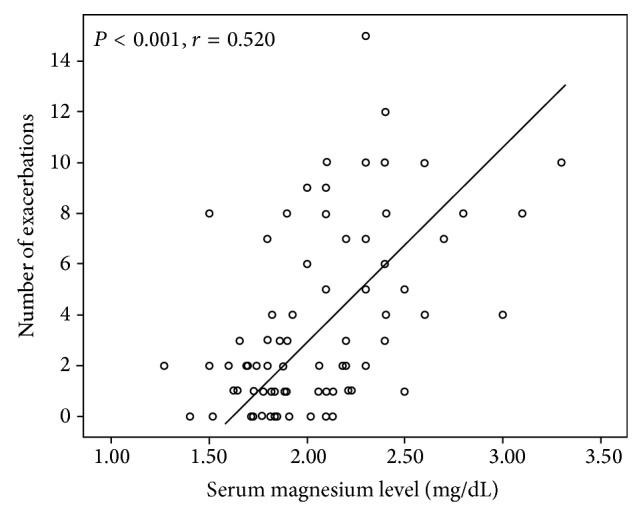
Scatterplot showing the positive correlation between number of exacerbations and serum magnesium level.

On Spearman correlation analysis the number of exacerbations during one year follow-up was negatively correlated with predicted FEV1% (*P* = 0.001), total protein (*P* = 0.024), globulin (*P* = 0.001), creatinine (*P* = 0.001), and uric acid levels (*P* = 0.036) and positively correlated with serum magnesium level (*P* < 0.001) (Figure 1) and platelet count (*P* = 0.043). According to linear regression analysis predicted FEV1% (beta = −0,227, *P* = 0.011), serum magnesium (beta = 0.431, *P* < 0.001), and globulin (beta = −0.250, *P* = 0.006) levels were independent predictors of number of exacerbations (Table 2).

**Figure 2:**
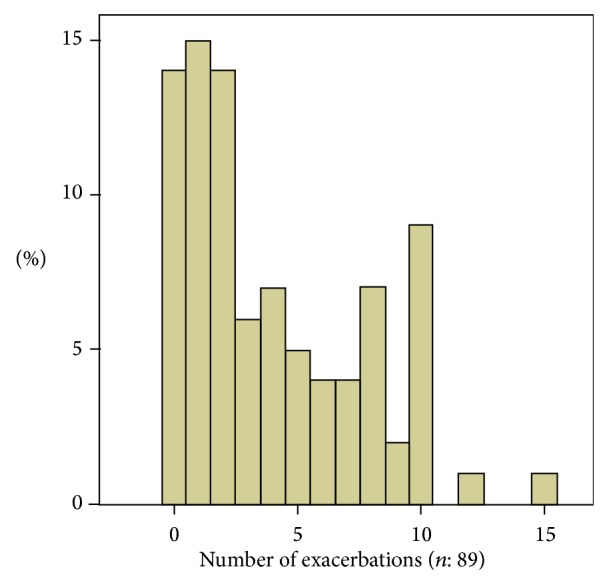
Histogram showing distribution of exacerbations.

**Figure 3:**
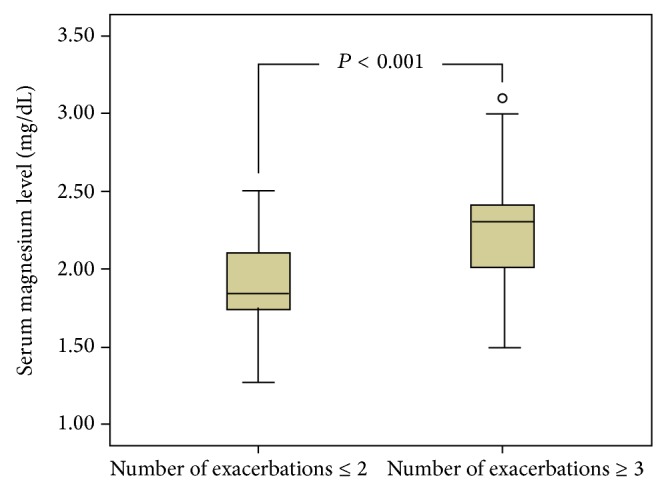
Box-plot showing the variation in magnesium levels between frequent and rare exacerbation groups.

The mean number of exacerbations in 1 year was 4.0 ± 3.6 (range 0–15). The distribution of exacerbations is shown in Figure 2. Patients were divided into two groups, those with a COPD-AE less than 3 and those with COPD-AE ≥ 3 per year. Predicted FEV% (*P* = 0.001), blood glucose (*P* < 0.001), creatinine (*P* < 0.001), uric acid (*P* = 0.021), and total protein (*P* = 0.006) levels were lower in the group with ≥3 exacerbations compared to those with ≤2, while platelet count (*P* = 0.028) and serum magnesium levels (*P* < 0.001) were higher (Table 3, Figure 3).

**Figure 4:**
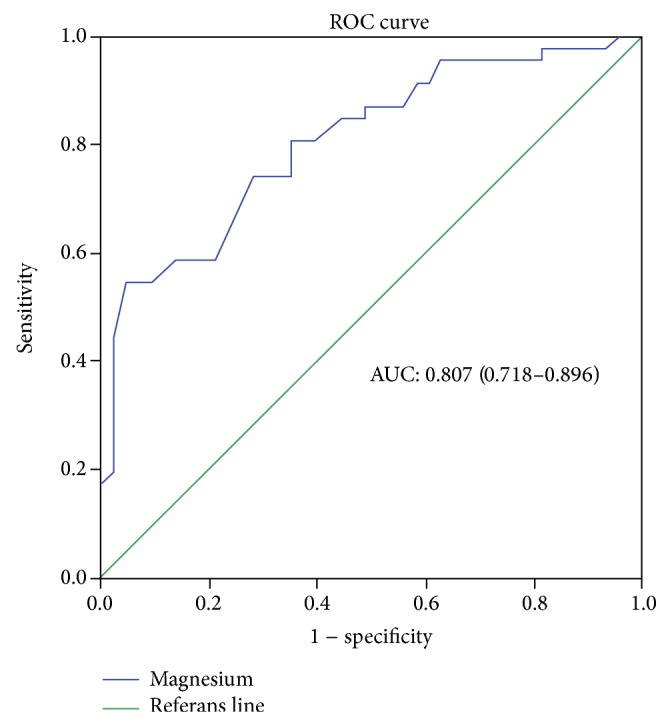
ROC curve analysis of serum magnesium level on frequency of COPD-AE.

As seen in ROC curve analysis serum magnesium level is a valuable predictor of frequent exacerbations in COPD (Figure 4). Area under curve (AUC) was determined at =0.807 (0.718–0.896), at a cut-off value of 2.26 mg/dL, and serum magnesium predicted the occurrence of 3 attacks or more per year with a sensitivity of 54.3% and specificity of 95.3%.

## 4. Discussion

Few studies have investigated the factors giving rise to acute exacerbations in patients with COPD. In this study, predicted FEV1%, serum globulin, and serum magnesium levels were identified as independent predictors of acute exacerbations of COPD and serum magnesium level was the most significant of these predictors.

A negative correlation was determined between predicted FEV1% and number of exacerbations. That is an expected outcome. Frequent exacerbations occur as the disease progresses. A low FEV1% has been associated with frequent exacerbations [12–14]. Coa et al. [6] determined a correlation between FEV1% <50% and frequent hospitalization due to acute exacerbations. Our study results also show that number of attacks rises as predicted FEV1% decreases. Another independent predictor of acute exacerbations in our study was serum globulin level. Total protein consists of albumin and globulin. No correlation between albumin level and exacerbation frequency was observed in this study. However, a negative correlation was determined between globulin level and COPD-AE. Globulin proteins consist of four groups, alpha 1, alpha 2, beta, and gamma. Subgroups were not investigated in this study. It is therefore not possible to state whether or not subgroup globulin levels may have an association frequency of COPD-AE. Alpha 1-antitrypsin deficiency is known to be a significant genetic risk factor for COPD [15].

The most important finding of this study is the positive correlation between serum magnesium level during acute exacerbation and annual number of COPD-AE. Number of attacks increased in association with serum magnesium levels. This is a significant finding. To the best of our knowledge, this is the first time that this correlation has been identified. Although it has not been proved, it is generally believed that due to its bronchodilating effect, a decreased level of magnesium increases COPD exacerbations. Unfortunately, we have not measured serum magnesium levels during stable period. Limited numbers of studies have investigated the relationship between magnesium and COPD-AE. Corradi et al. [16] determined higher serum magnesium levels in periods of COPD-AE compared to stable periods. A negative correlation was shown between magnesium and predicted FEV1%. However, they did not search the correlation between frequency of COPD-AE and magnesium levels. In a retrospective study, Aziz et al. [17] compared a group of COPD patients in the stable period with a different group of COPD patients during acute exacerbation. Serum magnesium levels were higher in the stable period group compared to the COPD-AE group. Fiaccadori et al. [18] could not find a significant difference in serum magnesium levels of COPD-AE in the ICU and a control healthy group. Contrary to our finding Bhatt et al. [19] found a reverse correlation between serum magnesium level and frequency of COPD-AE. However, there are important problems requiring criticism in that study; contrary to expectation, it is noteworthy that FEV1% and the frequencies of influenza and pneumococcal vaccination were higher in the group with frequent COPD-AE compared to the rare COPD-AE group.

Our study has certain limitations. First, the study population consisted almost entirely of males. Our study results may not therefore reflect all individuals with COPD-AE. However, this was not planned beforehand; the patients were included consecutively. We attribute this to COPD being more common in males than females in Turkish society. Second, there were no patients with mild (Stage 1) COPD in our study group. Third, serum magnesium levels were measured in the acute exacerbation period on admission to hospital. Magnesium levels in the stable period were not examined. Magnesium levels could not, therefore, be compared between the stable and COPD-AE patients. We speculate that the increased Mg levels in frequent exacerbation phenotypes of COPD may be from the body's attempt to optimize bronchodilation. Whether magnesium levels define a new phenotype of COPD or explain existing high exacerbation frequency phenotypes of COPD will require additional studies.

In conclusion, serum magnesium level during acute exacerbation period might be correlated with frequency of COPD-AE. The mechanism involved is as yet unclear. Larger and further studies are needed to search this association.

## Figures and Tables

**Table 1:** The demographic characteristics of patients.

Parameters	*n* = 89 (100%)
Age (year, mean ± sd) (range)	70.4 ± 7.8 (47–90)
Sex	
Female (%)	1 (1%)
Male (%)	88 (99%)
BMI (kg/m^2^)	25.5 ± 5.1
Smoking status	
Smoker	19 (21%)
Ex-smoker	69 (78%)
Nonsmoker	1 (1%)
Comorbidities	
HT	22 (25%)
Type 2 DM	13 (15%)
CAD	10 (11%)
BPH	6 (7%)
Bronchiectasis	4 (5%)
COPD stage	
Stage I	0 (0%)
Stage II	18 (20%)
Stage III	49 (55%)
Stage IV	22 (25%)

*n*: the number of patients; BMI: body mass index; mean ± sd: mean ± standard deviation; HT: hypertension; Type 2 DM: type 2 diabetes mellitus; CAD: coronary artery disease; BPH: benign prostatic hypertrophy.

**Table 2:** Linear regression analysis showing factors affecting frequency of COPD-AE.

	Standardized coefficients (Beta)	*t*	*P* value
Predicted FEV1%	−0.227	−2.590	0.011^*^
Serum creatinine	0.050	0.499	0.619
Serum uric acid	−0.040	−0.392	0.696
Serum protein	0.005	0.032	0.975
Serum globulin	−0.250	−2.839	0.006^*^
Serum magnesium	0.431	4.929	<0.001^*^
Platelet count	0.119	1.329	0.188

^*^Statistically significant.

**Table 3:** Comparison of variables between groups established on the basis of number of exacerbations.

	COPD-AE ≤ 2/year (*n*: 43)	COPD-AE ≥ 3/year (*n*: 46)	*P* value
Age, years (mean ± sd)	72 ± 8	69 ± 7	0.181
Sex, (M/F)	43/0	45/1	1.000
BMI, body mass index	25.6 ± 3.7	24.2 ± 4.5	0.301
Smoking, pack-year	65 ± 34	63 ± 35	0.815
FEV1, % of predicted	45 ± 16	34 ± 10	0.001^*^
FVC, % of predicted	58 ± 19	51 ± 12	<0.001^*^
pH	7.39 ± 0.06	7.40 ± 0.05	0.262
PaO2, mmHg	58 ± 10	57 ± 10	0.740
PaCO2, mmHg	42 ± 8	42 ± 11	0.711
Hb, gr/dL	14.2 ± 2.3	14.3 ± 1.7	0.961
Glucose, mg/dL	133 ± 47	106 ± 53	<0.001^*^
Platelet, ×10^3^	243 ± 65	280 ± 88	0.028^*^
Urea, mg/dL	44 ± 17	46 ± 19	0.582
Creatinine, mg/dL	1.03 ± 0.31	0.86 ± 0.23	<0.001^*^
Uric acid, mg/dL	6.5 ± 1.6	5.7 ± 1.7	0.021
Magnesium, mg/dL	1.88 ± 0.26	2.27 ± 0.37	<0.001^*^
Protein, g/dL	7.3 ± 0.7	6.9 ± 0.8	0.006^*^
Albumin, g/dL	3.8 ± 0.5	3.7 ± 0.4	0.659
Globülin, g/dL	3.6 ± 0.6	3.1 ± 0.7	0.004^*^
TSH, IU/mL	0.75 ± 1.28	0.69 ± 0.73	0.078

*n*: the number of patients; M: male; F: female; FEV1: expiratory volume percent at the first second of forced vital capacity over expected value; PaO2: arterial partial oxygen pressure; PaCO2: arterial partial carbon dioxide pressure.

^*^Statistically significant.
